# Diagnostic value of carbohydrate antigen 50 in biliary tract cancer: A large‐scale multicenter study

**DOI:** 10.1002/cam4.7388

**Published:** 2024-06-26

**Authors:** Yong‐Shuai Wang, Wei Wang, Shen‐Yu Zhang, Wei Cai, Rui‐Peng Song, Tao Mei, Wei Wang, Feng Zhang, Fei‐Yu Qi, Sai Zhang, Yan Liu, Hao‐Ran Li, Peng Ji, Miao Gao, Hua‐Chuan Song, Huan‐Zhang Yao, Fan‐Zheng Meng, Zheng Lu, Ji‐Zhou Wang, Lian‐Xin Liu

**Affiliations:** ^1^ Department of Hepatobiliary Surgery, Centre for Leading Medicine and Advanced Technologies of IHM, The First Affiliated Hospital of USTC, Division of Life Sciences and Medicine University of Science and Technology of China Hefei Anhui China; ^2^ Department of Medical Oncology, The First Affiliated Hospital of USTC, Division of Life Sciences and Medicine University of Science and Technology of China Hefei Anhui China; ^3^ Department of Physical Examination Center, The First Affiliated Hospital of USTC, Division of Life Sciences and Medicine University of Science and Technology of China Hefei Anhui China; ^4^ Department of Pathology, The First Affiliated Hospital of USTC, Division of Life Sciences and Medicine University of Science and Technology of China Hefei Anhui China; ^5^ Department of Hepatobiliary Surgery The First Affiliated Hospital of Bengbu Medical University Bengbu Anhui China; ^6^ Anhui Province Key Laboratory of Hepatopancreatobiliary Surgery Hefei Anhui China; ^7^ Anhui Provincial Clinical Research Center for Hepatobiliary Diseases Hefei Anhui China

**Keywords:** carbohydrate antigen 19‐9 (CA19‐9), carbohydrate antigen 50 (CA50), diagnostic biomarker, diagnostic model, intrahepatic cholangiocarcinoma (iCCA)

## Abstract

**Background:**

To date, carbohydrate antigen 19‐9 (CA19‐9) and carcinoembryonic antigen (CEA) have been widely used for the screening, diagnosis and prediction of biliary tract cancer (BTC) patients. However, few studies with large sample sizes of carbohydrate antigen 50 (CA50) were reported in BTC patients.

**Methods:**

A total of 1121 patients from the Liver Cancer Clin‐Bio Databank of Anhui Hepatobiliary Surgery Union between January 2017 and December 2022 were included in this study (673 in the training cohort and 448 in the validation cohort): among them, 458 with BTC, 178 with hepatocellular carcinoma (HCC), 23 with combined hepatocellular‐cholangiocarcinoma, and 462 with nontumor patients. Receiver operating characteristic (ROC) curves and decision curve analysis (DCA) were used to evaluate the diagnostic efficacy and clinical usefulness.

**Results:**

ROC curves obtained by combining CA50, CA19‐9, and AFP showed that the AUC value of the diagnostic MODEL 1 was 0.885 (95% CI 0.856–0.885, specificity 70.3%, and sensitivity 84.0%) in the training cohort and 0.879 (0.841–0.917, 76.7%, and 84.3%) in the validation cohort. In addition, comparing iCCA and HCC (235 in the training cohort, 157 in the validation cohort), the AUC values of the diagnostic MODEL 2 were 0.893 (95% CI 0.853–0.933, specificity 96%, and sensitivity 68.6%) in the training cohort and 0.872 (95% CI 0.818–0.927, 94.2%, and 64.6%) in the validation cohort.

**Conclusion:**

The model combining CA50, CA19‐9, and AFP not only has good diagnostic value for BTC but also has good diagnostic value for distinguishing iCCA and HCC.

## 1 INTRODUCTION

Biliary tract cancer (BTC) comprises a group of malignancies originating in the epithelium of the biliary tract and is a relatively rare cancer worldwide. However, it is prevalent in Asia.^1^, ^2^ A lack of robust screening measures, no specific clinical symptoms in the early stage, and late diagnosis (unresectable to metastatic) contribute to poor overall survival in BTCs.^3^ The National Comprehensive Cancer Network (NCCN) Clinical Practice Guidelines in Hepatobiliary Cancers also note the lack of effective tumor markers for BTC.^4^

BTCs are classified into five types, including intrahepatic cholangiocarcinoma (iCCA), perihilar cholangiocarcinoma (pCCA), distal cholangiocarcinoma (dCCA), vater ampulla carcinoma (VPC), and carcinoma of the gallbladder (GBC), based on anatomical location to help develop a therapeutic treatment plan clinically.^5^ Among them, a definitive diagnosis of iCCA requires liver biopsy analysis because it is difficult to distinguish iCCA from hepatocellular carcinoma (HCC) or combined hepatocellular‐cholangiocarcinoma (CHC) by imaging alone.^6^ Therefore, to distinguish between iCCA and HCC and CHC patients before treatment, the development of efficient tumor biomarkers remains pivotal.

Traditionally, there are several BTC biomarkers that have been recommended by the NCCN guidelines to screen for the disease early, diagnose the disease, set prognoses, and determine the efficacy of treatments, such as carbohydrate antigen 19‐9 (CA19‐9) and carcinoembryonic antigen (CEA).^4^, ^7^, ^8^, ^9^, ^10^, ^11^ The sensitivity of CA19‐9 and CEA is high, but their specificity is poor. Other investigators have questioned the utility of these tumor markers in predicting the development of cholangiocarcinoma or other pancreaticobiliary tumors due to a high false positive rate.^12^, ^13^, ^14^ Thus, exploring and discovering novel, specific, effective biomarkers will facilitate the identification of BTCs and other biliary tract malignancies.

Previously, carbohydrate antigen 50 (CA50) was reported to be a ganglioside glycoprotein and played an important clinical role in the screening and diagnosis of gastrointestinal malignancies, particularly pancreatic and colon cancer.^15^, ^16^, ^17^, ^18^ Despite extensive research on the screening and diagnostic value of CA50, its powerful evidence and data in BTC or CHC patients have rarely been explored.^19^, ^20^ To date, the exact clinical value of CA50 in BTCs remains unclear. Whether CA50 was more effective in combination with other tumor markers was also unclear.

Thus, we designed a large‐scale multicenter study to explore the screening and diagnostic values of CA50, CA19‐9, CEA, and α‐fetoprotein (AFP) in BTC patients and to address the following questions. First, we clarified the relationship among serum CA50, CA19‐9, AFP, and CEA levels and BTC patients, as well as its optimal threshold, sensitivity, specificity, accuracy, and so on. Second, we found that CA50 was significantly correlated with CA19‐9 in BTC patients. However, CA50 is a neglected tumor marker with high specificity. Furthermore, we combined CA50, CA19‐9 and AFP to build a clinical diagnostic model and dynamic nomogram to help differentiate iCCA patients from HCC patients before treatment.

## 2 METHODS

### 2.1 Study Design

**FIGURE 1:**
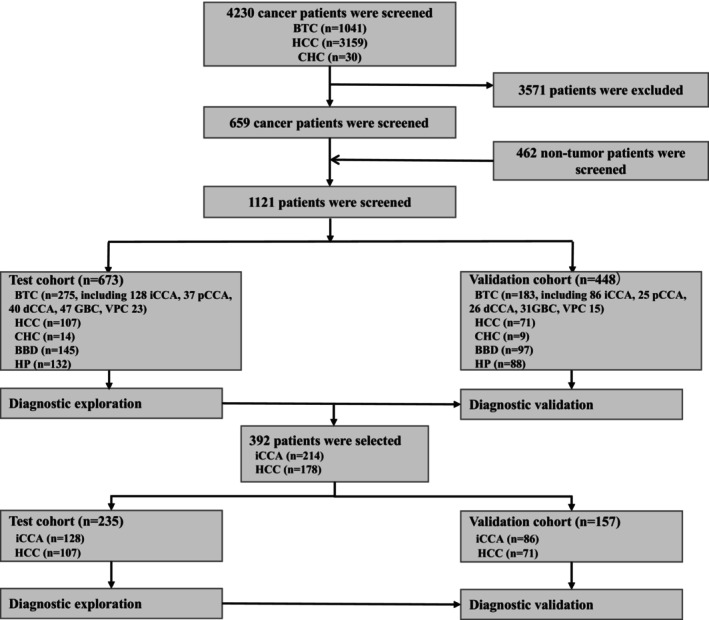
Flow chart of the study.

The clinical data of 4230 cancer patients from two centers of the Liver Cancer Clin‐Bio Databank of Anhui Hepatobiliary Surgery Union (LCCBD_AHSU) and 462 nontumor patients from the Physical Examination Center of the First Affiliated Hospital of the University of Science and Technology in China were collected from January 2017 to December 2022. LCCBD_AHSU was collected from a prospectively maintained database and reviewed retrospectively. Among these, 1121 patients were included, and the remaining 3571 patients who met the exclusion criteria were excluded. Then, 458 patients with BTC, 178 patients with HCC, 220 healthy people (HP), 23 patients with CHC, and 242 patients with benign biliary disease (BBD) were collected. A total of 458 patients with BTC were identified, of which 405 (88.4%) were surgically resected and 53 (11.6%) were examined by pathological liver biopsy. The criteria for HP are the absence of gastrointestinal and hepatobiliary diseases and normal serological and biochemical tests, blood glucose, and lipids. All patients were randomly divided into a training cohort and a validation cohort at a ratio of 6:4 (673: 448). In addition, based on the tumor location, BTCs were divided into five categories, including 214 cases of iCCA, 62 cases of pCCA, 66 cases of dCCA, 78 cases of GBC, and 38 cases of VPC. To differentiate iCCA patients from HCC patients before treatment, 392 patients were selected and were also randomly divided at a 6:4 ratio (235:157). The study protocol was approved by the Ethics Committee (ID: 2023‐KY‐24). Due to the retrospective nature of the study, the demand for obtaining a written informed consent form that the patient signed was waived (Figure 1). This trial was registered in the Chinese Clinical Trial Registry (ChiCTR), ChiCTR2300069682.

### 2.2 Inclusion and exclusion criteria

The inclusion criteria were as follows: (1) cancer patients diagnosed with BTC, HCC, or CHC by pathological diagnosis; (2) patients receiving AFP, CA19‐9, CEA, or CA50 examinations; and (3) patients receiving imaging examinations.

The exclusion criteria were as follows: (1) lack of the serum tumor biomarkers or imaging training data that were analyzed in this study; and (2) missing the other clinical data that were analyzed in the study; (3) cancer patients had metastases at other locations.

The enrolled patients were required to meet all three inclusion criteria, and patients meeting any of the exclusion criteria were excluded.

### 2.3 Tumor marker analysis

The serum tumor marker levels were examined by an automatic CL‐6000i chemiluminescence immunoassay (CLIA) analyzer from Mindray Co., Ltd. (Mindray Industries Biomedical Engineering), Shenzhen, China.

Due to the limitations of laboratory reports, the serological results of some patients were reported as follows: CA50 > 500 U/mL, CA19‐9 > 1000 U/mL, AFP > 1200 ng/mL, and CEA > 1000 ng/mL. In addition, the results of the above patients were statistically analyzed using the maximum critical value of their reports. The level of tumor markers was normally displayed in the table. Due to the large partial maximum value, logarithmic transformation was taken in the figure considering the esthetics of the picture.

### 2.4 Statistical analysis

The statistical analyses were performed using IBM‐SPSS statistics (Version 26) and R language (Version 4.2.2). Normally distributed data are described as the mean ± standard deviation (SD). Skewed distribution data are described as medians (min, max). Normally distributed data combined with variance homogeneity were compared between multiple groups by *ANOVA*. Skewed distribution data or heterogeneity of variance were compared between multiple groups by the Kruskal–Wallis *H* test. The correlation analysis was compared between two groups by Spearman nonparametric linear analysis. The level of tumor markers was normally displayed in the table. Due to the large partial maximum value, logarithmic transformation was taken in the figure considering the esthetics of the picture. Receiver operating characteristic (ROC) curves were constructed to assess the sensitivity, specificity, accuracy, positive predictive value (PPV), negative predictive value (NPV), positive likelihood ratio (LR), negative LR and respective areas under the curves (AUCs) with 95% CIs. Decision curve analysis (DCA) and a nomogram were used to evaluate the clinical usefulness of the model. The diagnostic model was constructed by logistic regression and included CA50, CA19‐9 and AFP.

MODEL 1: logit = −1.83862 + 0.02204*CA50 + 0.01214*CA199–0.00245*AFP.

MODEL 2: logit = −0.75580 + 0.00766*CA50 + 0.01554*CA19.9–0.00999*AFP.

*p* < 0.05 was considered statistically significant.

## 3 RESULTS

### 3.1 Levels of tumor biomarkers in the BTC, HCC, CHC, BBD, and HP groups

**FIGURE 2:**
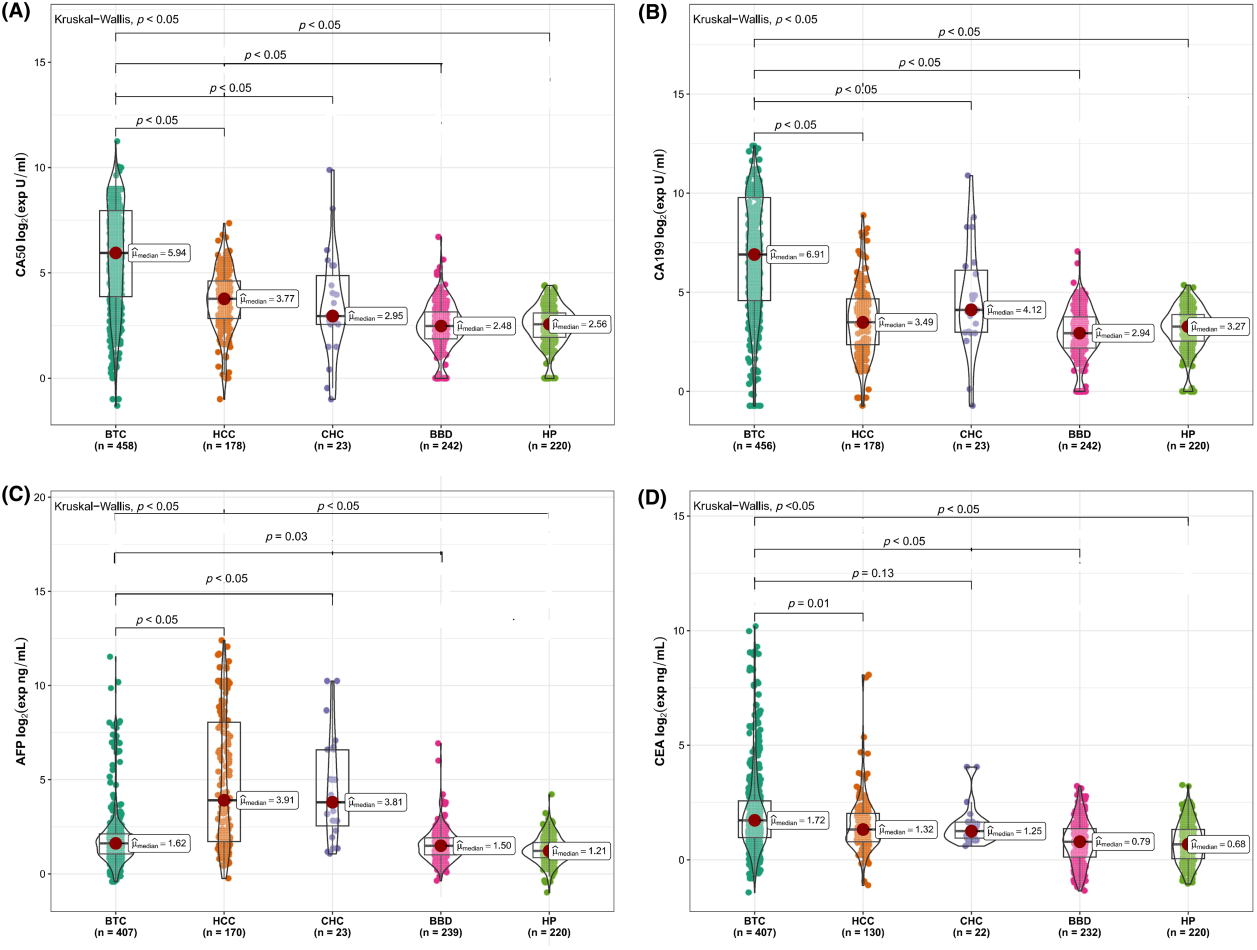
Differential expression levels of four tumor markers in the BTC, HCC, CHC, BBD, and HP groups. (A) Levels of serum CA50 in different groups; (B) Levels of serum CA19‐9 in different groups; (C) Levels of serum AFP in different groups; (D) Levels of serum CEA in different groups. AFP, α‐fetoprotein; BBD, benign biliary‐liver diseases; BTC, biliary tract cancer; CA19‐9, carbohydrate antigen 19‐9; CA50, carbohydrate antigen 50; CEA, carcinoembryonic antigen; CHC, combined hepatocellular‐cholangiocarcinoma; HCC, hepatocellular carcinoma; HP, healthy people.

To investigate the expression levels of different tumor markers in different types of populations, the preoperative expression levels of CA50, CA19‐9, AFP, and CEA in BTC patients were measured and compared with those of non‐BTC controls (Figure 2). The median levels of serum CA50 in the BTC, HCC, CHC, BBD, and HP groups were 61.5 U/mL, 13.6 U/mL, 7.7 U/mL, 5.6 U/mL and 5.9 U/mL, respectively. The median levels of serum CA19‐9 in the BTC, HCC, CHC, BBD, and HP groups were 120.3 U/mL, 11.2 U/mL, 17.3 U/mL, 7.7 U/mL and 9.7 U/mL, respectively. The median levels of serum AFP in the above groups were 3.1 ng/mL, 15.1 ng/mL, 14.0 ng/mL, 2.8 ng/mL and 2.3 ng/mL, respectively. The median levels of serum CEA in the above groups were 3.3 ng/mL, 2.5 ng/mL, 2.4 ng/mL, 1.7 ng/mL and 1.6 ng/mL, respectively (Table 1, Figure 2). The levels of serum CA50, CA19‐9, and CEA in the BTC patients were higher than those in the HCC, CHC, BBD, and HP patients, and the difference was significant (*p* < 0.001) (Table 1).

**TABLE 1:** Levels of tumor markers in the BTC, HCC, CHC, BBD, and HP groups.

	BTC (*n* = 458)	HCC (*n* = 178)	CHC (*n* = 23)	BBD (*n* = 242)	HP (*n* = 220)	*p*
Median CA50 (min, mix), U/ml	61.5 (0.4–2412.2)	13.6 (0.5–163.7)	7.7 (0.5–939.2)	5.6 (1.0–104.2)	5.9 (1.0–21.1)	<0.001
Median CA19‐9 (min, mix), U/ml	120.3 (0.6–5355.0)	11.2 (0.6–472.8)	17.3 (0.6–1888.5)	7.7 (1.0–133.5)	9.7 (1.0–41.1)	<0.001
Median AFP (min, mix), ng/mL	3.1 (0.8–2933.0)	15.1 (0.8–5400.0)	14.0 (2.1–1210.0)	2.8 (0.8–120.8)	2.3 (0.5–18.6)	<0.001
Median CEA (min, mix), ng/mL	3.3 (0.4–1160.3)	2.5 (0.5–268.1)	2.4 (1.5–16.5)	1.7 (0.4–9.3)	1.6 (0.5–9.5)	<0.001

Abbreviations: AFP, α‐fetoprotein; BBD, benign biliary‐liver diseases; BTC, biliary tract cancer; CA19‐9, carbohydrate antigen 19‐9; CA50, carbohydrate antigen 50; CEA, carcinoembryonic antigen; CHC, combined hepatocellular‐cholangiocarcinoma; HCC, hepatocellular carcinoma; HP, healthy people.

**FIGURE 3:**
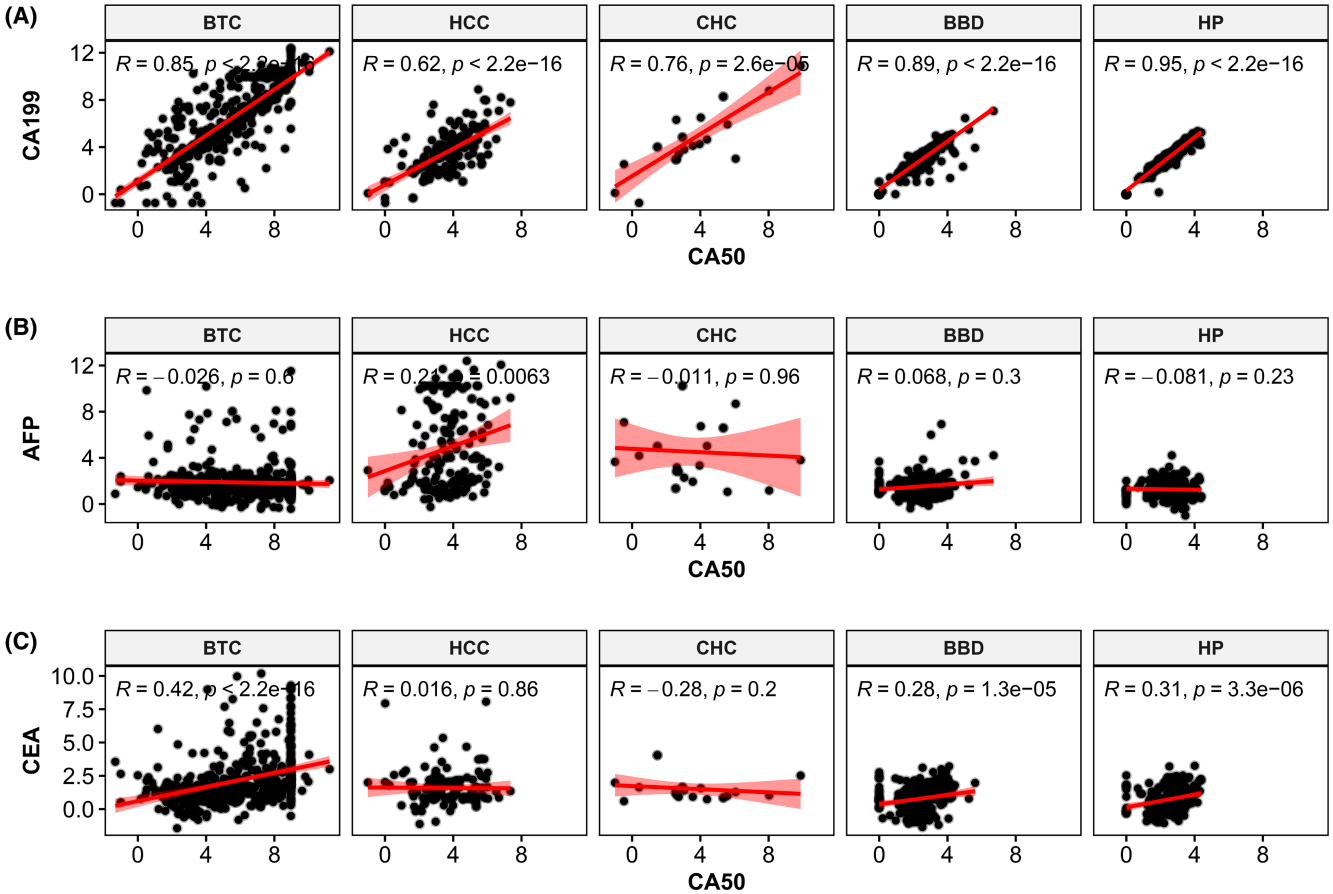
Correlation analysis between serum CA50 and other tumor markers. (A) Correlation of CA50 versus CA19‐9; (B) Correlation of CA50 versus AFP; (C) Correlation of CA50 versus CEA. AFP, α‐fetoprotein; BBD, benign biliary‐liver diseases; BTC, biliary tract cancer; CA19‐9, carbohydrate antigen 19‐9; CA50, carbohydrate antigen 50; CEA, carcinoembryonic antigen; CHC, combined hepatocellular‐cholangiocarcinoma; HCC, hepatocellular carcinoma; HP, healthy people.

In addition, correlation analysis of CA50 with other tumor markers is presented in Figure 3. The serum CA50 was significantly correlated with serum CA19‐9. The *R*‐values in BTC, HCC, CHC, BBD, and HP were 0.85, 0.62, 0.76, 0.89, and 0.95, respectively (Figure 3A). However, serum CA50 was not significantly correlated with serum AFP or CEA, and the *R*‐values were all small in the above patients (Figure 3B,C).

Therefore, the levels of serum CA50, CA19‐9, and CEA in BTC patients were higher than those in HCC, CHC, BBD, and HP patients. In addition, the levels of serum CA50 and CA19‐9 were significantly correlated in BTC, CHC, BBD, and HP patients.

### 3.2 Diagnostic values of serum tumor markers and the dynamic model in BTC patients

A total of 1121 persons were randomly divided into a training cohort and a validation cohort at a ratio of 6:4 (673:448). There were no significant statistical differences in age, sex, different levels of serum tumor markers, maximum tumor, or study population between the training cohort and the validation cohort (Table 2).

**TABLE 2:** Characteristics of the study population in both training cohort and validation cohort (BTC vs. HCC + CHC + BBD + HP).

Characteristic	Training cohort (*n* = 673)	Validation cohort (*n* = 448)	*p*
Mean Age (±SD), years	56.4 (13.5)	56.6 (14.3)	0.766
Sex, *n* (%)			0.922
Male	361 (53.7%)	242 (54.0%)	
Female	311 (46.3%)	206 (46.0%)	
Median CA50 (min, mix), U/ml	11.2 (0.4–1050.7)	9.8 (0.5–2412.2)	0.233
Median CA19‐9 (min, mix), U/ml	15.8 (0.6–5355.0)	15.0 (0.6–4421.0)	0.232
Median AFP (min, mix), ng/mL	3.1 (0.7–4277.9)	2.9 (0.5–5400.0)	0.117
Median CEA (min, mix), ng/mL	2.3 (0.4–543.0)	2.2 (0.5–1160.3)	0.231
Median Maximum tumor diameter (min, mix), mm	37.0 (1.0–170.0)	40.0 (4.0–173.0)	0.553
Study population, *n* (%)			1.000
BTC	275 (40.9%)	183 (40.8%)	
HCC	107 (15.9%)	71 (15.8%)	
CHC	14 (2.1%)	9 (2.0%)	
BBD	145 (21.5%)	97 (21.7%)	
HP	132 (19.6%)	88 (19.6%)	

Abbreviations: AFP, α‐fetoprotein; BBD, benign biliary‐liver diseases; BTC, biliary tract cancer; CA19‐9, carbohydrate antigen 19‐9; CA50, carbohydrate antigen 50; CEA, carcinoembryonic antigen; CHC, combined hepatocellular‐cholangiocarcinoma; HCC, hepatocellular carcinoma; HP, healthy people.

**FIGURE 4:**
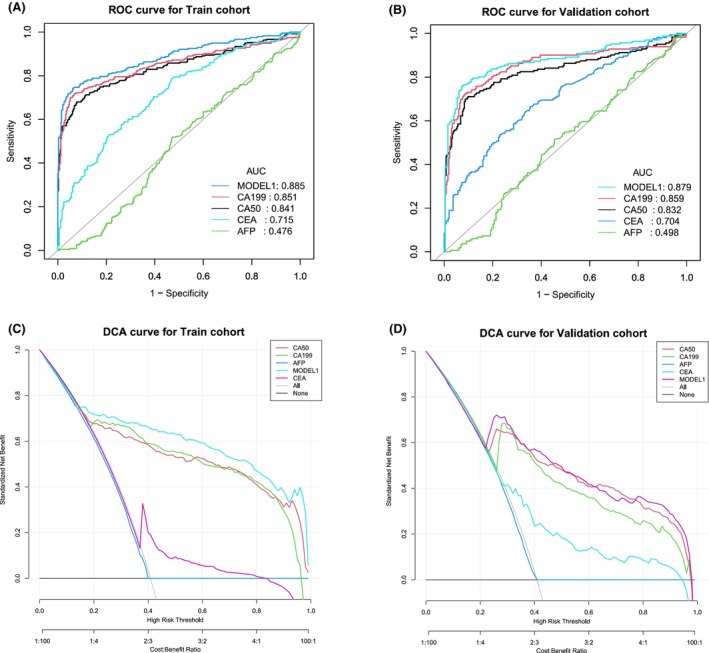
ROC results of different tumor markers and the model in the diagnosis of BTC. (A) ROC results of BTC versus HCC + HCC + BBD + HP in the training cohort; (B) ROC results of BTC versus HCC + HCC + BBD + HP in the validation cohort; (C) DCA results of BTC versus HCC + HCC + BBD + HP in the training cohort; (D) DCA results of BTC versus HCC + HCC + BBD + HP in the validation cohort. AFP, α‐fetoprotein; AUC, area under the curve; BBD, benign biliary‐liver diseases; BTC, biliary tract cancer; CA19‐9, carbohydrate antigen 19‐9; CA50, carbohydrate antigen 50; CEA, carcinoembryonic antigen; CHC, combined hepatocellular‐cholangiocarcinoma; HCC, hepatocellular carcinoma; HP, healthy people; LR, likelihood ratio; NPV, negative predictive value; PPV, positive predictive value; ROC, receiver operating characteristic curve. MODEL 1 (CA50 + CA19‐9 + AFP).

**FIGURE 5:**
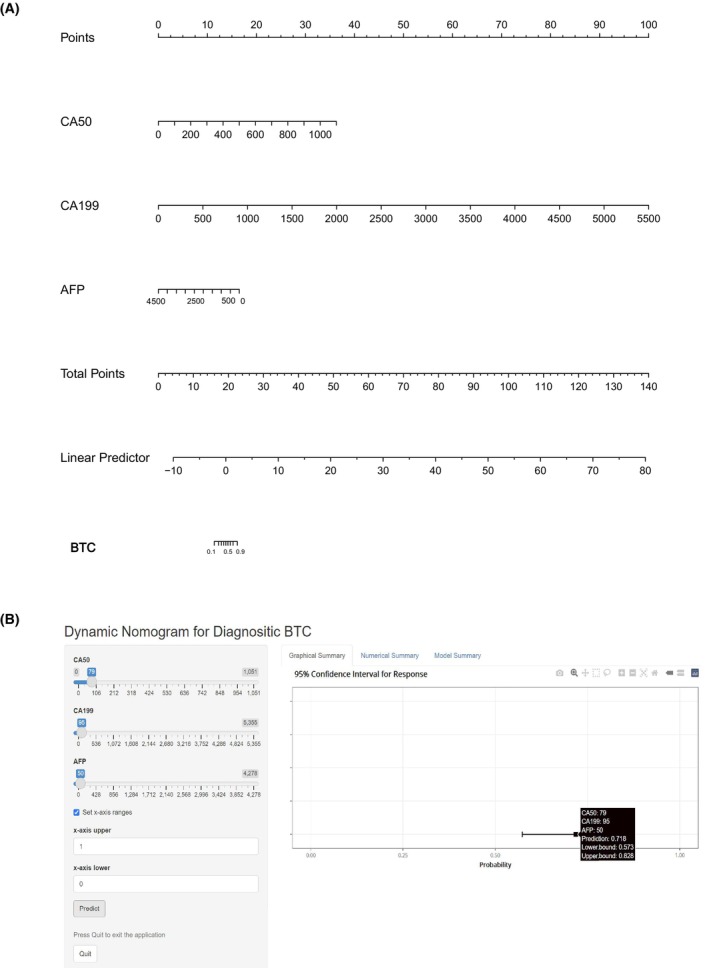
The nomogram of the MODEL 1 for BTC versus HCC + HCC + BBD + HP. (A) The static nomogram for BTC versus HCC + HCC + BBD + HP; (B) The dynamic nomogram for BTC versus HCC + HCC + BBD + HP.

In order to improve the screening efficacy and obtain a higher sensitivity, we chose the cut‐off value when the specificity was close to 70% as the best cut‐off value. Comparing BTC and non‐BTC, the best cut‐off value of the single serum CA50 level was 11.185 U/mL (training cohort: AUC 0.841, 95% CI 0.808–0.875, specificity 70.4%, sensitivity 80.0%; validation cohort: AUC 0.833, 95% CI 0.789–0.876, specificity 76.6%, sensitivity 78.1%). The best cut‐off value of CA19‐9 was 16.025 U/mL (training cohort: AUC 0.851, 95% CI 0.818–0.885, specificity 71.4%, sensitivity 79.6%; validation cohort: AUC 0.859, 95% CI 0.819–0.899, specificity 76.6%, sensitivity 82.4). The best cut‐off value of AFP was 4.850 ng/mL (training cohort: AUC 0.476, 95% CI 0.430–0.521, specificity 70.0%, sensitivity 21.2%; validation cohort: AUC 0.498, 95% CI 0.442–0.553, specificity 76.7%, sensitivity 19.3%). And the best cut‐off value of CEA was 2.625 ng/mL (training cohort: AUC 0.715, 95% CI 0.673–0.757, specificity 70.4%, sensitivity 60.1%; validation cohort: AUC 0.704, 95% CI 0.653–0.756, specificity 71.4%, sensitivity 56.7%) (Table 3 and Figure 4). The AUCs of serum CA19‐9 and CA50 were higher than those of AFP and CEA, and serum CA50 and CA19‐9 had a better specificity and positive LR. The accuracy, positive predictive value (PPV), negative predictive value (NPV), positive likelihood ratio (LR), and negative LR of the different tumor markers are also shown in Table 3. In addition, we built a clinical diagnostic model by combining CA50, CA19‐9 and AFP, and the AUCs of the MODEL 1 were 0.885 (training cohort: 95% CI 0.856–0.915, specificity 70.3%, sensitivity 84.0%) and 0.879 (validation cohort: 95% CI 0.841–0.917, specificity 76.7%, sensitivity 84.3%), which were higher than those of the above serum tumor markers alone (Figure 4A,B). The results of DCAs also indicated that the MODEL 1 could add more benefit than the “treat none” and “treat all” (Figure 4C,D). In MODEL 1, we used the following formula: logit = −1.83862 + 0.02204*CA50 + 0.01214*CA199–0.00245*AFP. The nomogram of the MODEL 1 is shown in Figure 5A, and the dynamic nomogram is shown in Figure 5B (https://diagnosticnomogrambtc.shinyapps.io/dynnomapp/).

**TABLE 3:** ROC results of different serum tumor markers in the diagnosis of BTC versus HCC + CHC + BBD + HP.

	*N*	AUC (95% CI)	Cutoff value	Specificity (%)	Sensitivity (%)	Accuracy	PPV (%)	NPV (%)	Positive LR	Negative LR
Training Cohort (BTC vs. HCC + CHC + BBD + HP)										
CA50	673	0.841 (0.808–0.875)	24.875	92.2	68.0	0.823	85.8	80.7	8.730	0.347
CA19‐9	672	0.851 (0.818–0.885)	31.680	93.2	71.9	0.845	88.0	82.8	10.598	0.302
AFP	631	0.476 (0.430–0.521)	3.105	52.6	51.9	0.526	40.3	63.9	1.093	0.916
CEA	602	0.715 (0.673–0.757)	3.230	78.6	53.1	0.683	62.6	71.2	2.475	0.597
MODEL 1	631	0.885 (0.856–0.915)	−0.944	93.9	74.3	0.864	88.2	85.5	12.070	0.274
Validation Cohort (BTC vs. HCC + CHC + BBD + HP)										
CA50	448	0.833 (0.789–0.876)	24.875	91.7	65.6	0.810	0.845	0.794	7.899	0.375
CA19‐9	447	0.859 (0.819–0.899)	31.680	92.5	69.8	0.832	0.864	0.817	9.246	0.327
AFP	428	0.498 (0.442–0.553)	3.105	57.3	46.4	0.530	0.407	0.628	1.085	0.937
CEA	409	0.704 (0.653–0.756)	3.230	80.4	48.8	0.677	0.625	0.701	2.490	0.637
MODEL 1	428	0.879 (0.841–0.917)	−1.129	92.8	75.9	0.862	0.869	0.859	10.467	0.260

*Note*: MODEL (CA50 + CA19‐9 + AFP).

Abbreviations: AFP, α‐fetoprotein; AUC, area under the curve; BBD, benign biliary‐liver diseases; BTC, biliary tract cancer; CA19‐9, carbohydrate antigen 19‐9; CA50, carbohydrate antigen 50; CEA, carcinoembryonic antigen; CHC, combined hepatocellular‐cholangiocarcinoma; HCC, hepatocellular carcinoma; HP, healthy people; LR, likelihood ratio; NPV, negative predictive value; PPV, positive predictive value; ROC, receiver operating characteristic curve.

Therefore, CA50 is a potential tumor marker for the screening and diagnosis of BTC patients. To distinguish BTC and non‐BTC patients, serum CA50 and CA19‐9 had a better specificity and positive LR than those of AFP and CEA. The AUCs of the MODEL 1 by combining CA50, CA19‐9, and AFP were higher than those of the single serum tumor markers.

### 3.3 Diagnostic values of different tumor markers and the dynamic model in iCCA patients

A total of 392 persons were randomly divided into a training cohort and a validation cohort at a ratio of 6:4 (235:157). There were not significant statistical differences in age, sex, different levels of serum tumor markers, maximum tumor, or study population between the training cohort and the validation cohort (Table 4).

**TABLE 4:** Characteristics of the study population in both training cohort and validation cohort (iCCA vs. HCC).

Characteristic	Training cohort (*n* = 235)	Validation cohort (*n* = 157)	*p*
Mean Age (±SD), years	61.7 (10.5)	61.0 (10.4)	0.538
Sex, *n* (%)			0.719
Male	150 (63.8%)	103 (65.6%)	
Female	85 (36.2%)	54 (34.4%)	
Median CA50 (min, mix), U/ml	22.3 (0.4–2412.2)	19.6 (0.5–500.0)	0.183
Median CA19‐9 (min, mix), U/ml	30.3 (0.6–5355.0)	25.4 (0.6–5353.0)	0.187
Median AFP (min, mix), ng/mL	3.9 (0.8–5400.0)	4.3 (0.8–3291.0)	0.705
Median CEA (min, mix), ng/mL	3.3 (0.5–1000.0)	3.3 (0.6–1160.3)	0.791
Median Maximum tumor diameter (min, mix), mm	230 (53.5)	154 (53.8)	0.932
Study population, *n* (%)			0.952
iCCA	128 (54.5%)	86 (54.8%)	
HCC	107 (45.5%)	71 (45.2%)	

Abbreviations: AFP, α‐fetoprotein; CEA, carcinoembryonic antigen; CA50, carbohydrate antigen 50; CA19‐9, carbohydrate antigen 19‐9; HCC, hepatocellular carcinoma; iCCA, intrahepatic cholangiocarcinoma.

**FIGURE 6:**
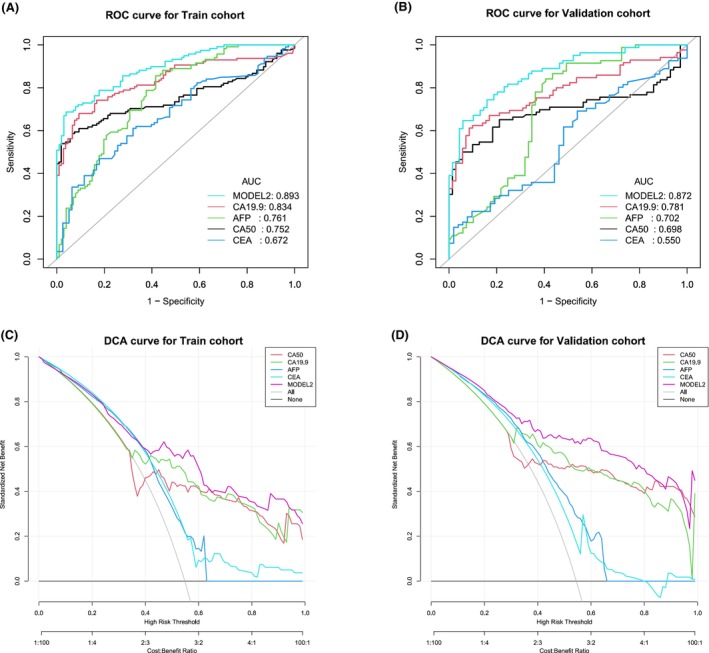
ROC results of different tumor markers and the model in the diagnosis of iCCA. (A) ROC results of iCCA versus HCC in the training cohort; (B) ROC results of iCCA versus HCC in the validation cohort; (C) DCA results of iCCA versus HCC in the training cohort; (D) DCA results of iCCA versus HCC in the validation cohort. AFP, α‐fetoprotein; AUC, area under the curve; BBD, benign biliary‐liver diseases; BTC, biliary tract cancer; CA19‐9, carbohydrate antigen 19‐9; CA50, carbohydrate antigen 50; CEA, carcinoembryonic antigen; CHC, combined hepatocellular‐cholangiocarcinoma; HCC, hepatocellular carcinoma; HP, healthy people; LR, likelihood ratio; NPV, negative predictive value; PPV, positive predictive value; ROC, receiver operating characteristic curve. MODEL 2 (CA50 + CA19‐9 + AFP).

**FIGURE 7:**
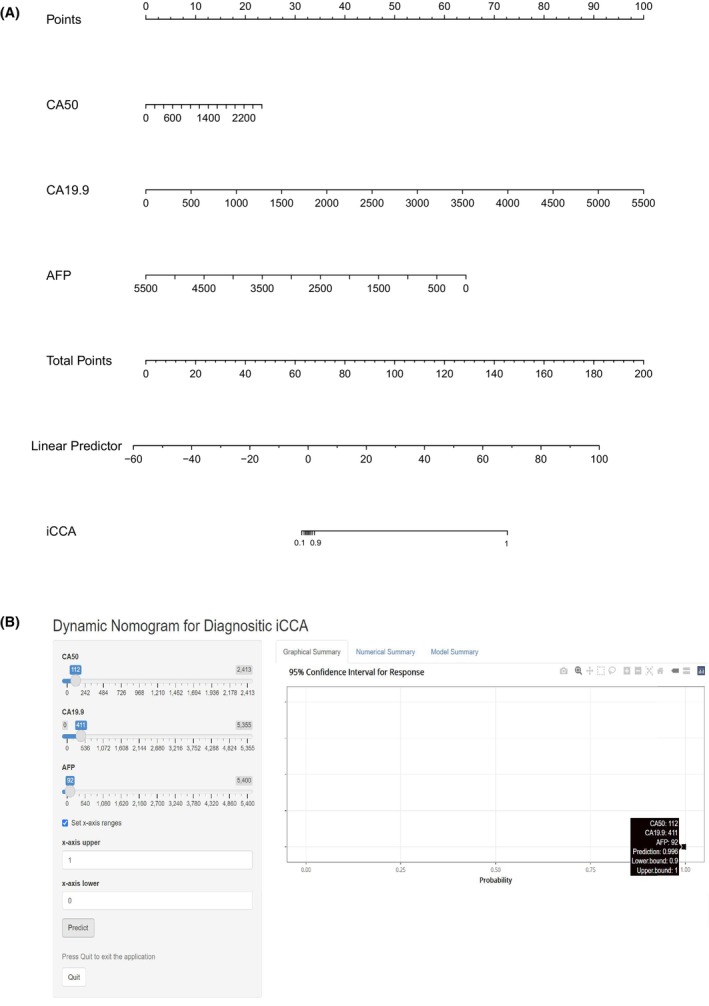
The nomogram of the MODEL for iCCA versus HCC. (A) The static nomogram for iCCA versus HCC; (B) The dynamic nomogram for iCCA versus HCC.

The best cutoff value is chosen as the cut‐off value at the maximum value of specificity plus sensitivity. Distinguishing iCCA and HCC, the best cutoff value of serum CA50 was 52.995 U/mL (training cohort: AUC 0.752, 95% CI 0.688–0.816, specificity 93.5%, sensitivity 58.6%; validation cohort: AUC 0.698, 95% CI 0.612–0.783, specificity 94.4%, sensitivity 48.8%). And the best cut‐off value of serum CA19‐9 was 52.700 U/mL (training cohort: AUC 0.834, 95% CI 0.781–9.887, specificity 90.7%, sensitivity 68.0%; validation cohort: AUC 0.781, 95% CI 0.708–0.854, specificity 91.6%, sensitivity 58.8%). The best threshold of AFP was 6.750 ng/mL (training cohort: AUC 0.761, 95% CI 0.697–0.825, specificity 58.4%, sensitivity 85.6%; validation cohort: AUC 0.702, 95% CI 0.614–0.790, specificity 59.4%, sensitivity 84.2%). The best threshold of CEA was 3.325 ng/mL (training cohort: AUC 0.672, 95% CI 0.595–0.748, specificity 68.0%, sensitivity 61.1%; validation cohort: AUC 0.550, 95% CI 0.450–0.651, specificity 53.9%, sensitivity 50.6%) (Table 5 and Figure 6). The accuracy, PPV, NPV, positive LR, and negative LR of the different tumor markers are also shown in Table 5. In addition, we also built a clinical diagnostic model by combining CA50, CA19‐9, and AFP, and the AUCs of the MODEL 2 were 0.893 (training cohort: 95% CI 0.853–0.933, specificity 96.0%, sensitivity 68.6%) and 0.872 (validation cohort: 95% CI 0.818–0.927, specificity 94.2%, sensitivity 64.6%), which were higher than those of the above tumor markers alone (Figure 6A,B). The results of DCAs also indicated that the MODEL 2 could add more benefit than the “treat none” and “treat all” (Figure 6C,D). In MODEL 2, we used the following formula: logit = −0.75580 + 0.00766*CA50 + 0.01554*CA19.9–0.00999*AFP. The nomogram of the MODEL is shown in Figure 7A, and the dynamic nomogram is shown in Figure 7B (https://diagnosticnomogramicca.shinyapps.io/dynnomapp/).

**TABLE 5:** ROC results of different serum tumor markers in the diagnosis of iCCA versus HCC.

	*N*	AUC (95% CI)	Cutoff value	Specificity (%)	Sensitivity (%)	Accuracy	PPV (%)	NPV (%)	Positive LR	Negative LR
Training Cohort (iCCA vs. HCC)										
CA50	235	0.752 (0.688–0.816)	52.995	93.5	58.6	0.745	0.915	0.654	8.957	0.443
CA19‐9	234	0.834 (0.781–0.887)	52.700	90.7	68.0	0.783	0.897	0.703	7.273	0.353
AFP	221	0.761 (0.697–0.825)	6.750	58.4	85.6	0.731	0.706	0.776	2.058	0.247
CEA	196	0.672 (0.595–0.748)	3.325	68.0	61.1	0.639	0.734	0.546	1.905	0.573
MODEL 2	221	0.893 (0.853–0.933)	0.389	96.0	68.6	0.813	0.953	0.724	17.333	0.327
Validation Cohort (iCCA vs. HCC)										
CA50	157	0.698 (0.612–0.783)	52.995	94.4	48.8	0.694	0.913	0.604	8.669	0.542
CA19‐9	157	0.781 (0.708–0.854)	52.700	91.6	58.8	0.737	0.893	0.650	6.961	0.450
AFP	149	0.702 (0.614–0.790)	6.750	59.4	84.2	0.713	0.759	0.759	2.074	0.267
CEA	128	0.550 (0.450–0.651)	3.325	53.9	50.6	0.519	0.631	0.412	1.097	0.917
MODEL 2	149	0.872 (0.818–0.927)	0.240	94.2	64.6	0.782	0.930	0.692	11.149	0.375

*Note*: MODEL (CA50 + CA19‐9 + AFP).

Abbreviations: AFP, α‐fetoprotein; AUC, area under the curve; BBD, benign biliary‐liver diseases; BTC, biliary tract cancer; CA19‐9, carbohydrate antigen 19‐9; CA50, carbohydrate antigen 50; CEA, carcinoembryonic antigen; CHC, combined hepatocellular‐cholangiocarcinoma; HCC, hepatocellular carcinoma; HP, healthy people; iCCA, intrahepatic cholangiocarcinoma; LR, likelihood ratio; NPV, negative predictive value; PPV, positive predictive value; ROC, receiver operating characteristic curve.

Therefore, compared to CA19‐9, AFP, and CEA, serum CA50 had a better specificity and positive LR in iCCA patients. The AUCs of the MODEL 2 by combining CA50, CA19‐9, and AFP were higher than those of the single serum tumor markers.

### 3.4 Clarification of the relationship between different tumor marker levels and BTC location

The BTCs were classified into five types, including iCCA, pCCA, dCCA, GBC, and VPC, based on their anatomic location. A total of 458 BTC patients were classified, 214 with iCCA, 62 with pCCA, 66 with dCCA, 78 with GBC, and 38 with VPC. The overall distribution of the different tumor markers in different locations of BTC is shown in Figure S1. The median levels of serum CA50 in the iCCA, pCCA, dCCA, GBC, and VPC patients were respectively 66.5 U/mL, 95.3 U/mL, 61.3 U/mL, 30.9 U/mL and 77.7 U/mL, respectively. The median levels of serum CA19‐9 in the iCCA, pCCA, dCCA, GBC, and VPC patients were respectively 134.4 U/mL, 231.9 U/mL, 62.1 U/mL, 62.5 U/mL, and 104.8 U/mL respectively (Table S1). The median levels of serum AFP in the iCCA, pCCA, dCCA, GBC, and VPC patients were respectively 3.2 U/mL, 3.4 U/mL, 2.9 U/mL, 2.4 U/mL, and 2.5 U/mL, respectively. The median levels of serum CEA in the above locations were 3.5 ng/mL, 3.7 ng/mL, 2.9 ng/mL, 2.3 ng/mL, and 3.5 ng/mL, respectively. The serum CA19‐9, AFP, and CEA values were significantly different at different locations of BTCs (*p* = 0.020, *p* = 0.026, *p* = 0.001; Figure S1 and Table S1).

Therefore, the levels of serum AFP, CA19‐9, and CEA were correlated with the location of BTCs. The serum CA50 and CA19‐9 levels were higher in pCCA than in iCCA, dCCA, GBC, and VPC.

### 3.5 Clarification of the relationship between different tumor marker levels and the degree of jaundice

Based on the total bilirubin levels (TBIL), patients with BTC were divided into five groups: no jaundice group (TBIL <17.1 μmol/L), recessive jaundice group (TBIL: 17.1–34.2 μmol/L), mild jaundice group (TBIL: 34.3–171.0 μmol/L), moderate jaundice group (TBIL: 172.0–342.0 μmol/L), and severe jaundice group (TBIL >342.0 μmol/L) (Figure S2). The median serum CA50 levels in the above five groups were 28.5 U/mL, 65.3 U/mL, 80.7 U/mL, 132.0 U/mL, and 312.8 U/mL, and the median serum CA19‐9 levels in the above five groups were 53.6 U/mL, 121.4 U/mL, 143.6 U/mL, 173.8 U/mL, and 522.9 U/mL. The serum levels of both CA50 and CA19‐9 increased with the severity of jaundice, and the difference was statistically significant (*p* < 0.001, *p =* 0.010). There was no significant difference between the serum CEA and AFP levels and the degree of jaundice in the different groups (*p =* 0.721, *p =* 0.583; Table S2 and Figure S2).

Therefore, the serum CA50 and CA19‐9 levels increased with the degree of jaundice.

### 3.6 Clarification of the relationship among different tumor marker levels and the degree of pathological pattern and AJCC staging

Based on the pathological examination results, patients with BTC were divided into four groups: poorly differentiated adenocarcinoma, moderately differentiated adenocarcinoma, highly differentiated adenocarcinoma, and other types (Figure S3). Other types included squamous cell carcinoma, adeno‐squamous carcinoma, neuroendocrine carcinoma, and so on. The median serum CA50 levels in the above four groups were 66.6 U/mL, 63.8 U/mL, 35.2 U/mL, and 46.3 U/mL, and the median serum CA19‐9 levels in the above four groups were 134.4 U/mL, 102.9 U/mL, 60.4 U/mL, and 110.1 U/mL. The serum levels of CA50 and CA19‐9 were highest in the poorly differentiated adenocarcinoma. The serum levels of CA19‐9 increased with the severity of jaundice (*p =* 0.048). There was no significant difference between the serum CA50, CEA, and AFP levels and the degree of jaundice in different groups (*p =* 0.358, *p =* 0.795, *p =* 0.294; Table S3 and Figure S3).

Based on the American Joint Committee on Cancer (AJCC) staging 8th edition in BTC, patients were divided into three groups: AJCC 0–1 staging (*n* = 316), AJCC 2 staging (*n* = 100), and AJCC 3–4 staging (*n* = 42) (Table S4). The median serum CA50 levels in the above three groups were 58.6 U/mL, 74.9 U/mL, and 182.8 U/mL. The serum levels of CA50 increased with the AJCC staging (*p =* 0.037). The median serum CA199 levels in the above three groups were 96.9 U/mL, 169.8 U/mL, and 223.4 U/mL, and the median serum AFP levels in the above three groups were 3.1 U/mL, 3.1 U/mL, and 2.8 U/mL. There was no significant difference between the serum CA199, CEA, and AFP levels and the AJCC staging in different groups (*p =* 0.171, *p =* 0.619, *p =* 0.070).

Therefore, the serum CA19‐9 levels were obviously higher in the poorly differentiated adenocarcinoma than in highly differentiated adenocarcinoma in BTCs. And the serum CA50 levels were obviously higher in the AJCC 3–4 staging than in the other AJCC staging.

## 4 DISCUSSION

Traditionally, many guidelines and studies have recommended CA19‐9 and CEA as screening and diagnostic markers for BTC or iCCA patients. Few studies have reported the potential diagnostic value of CA50 in these patients ^[19.20]^. Some studies have proven that altered glycosylation occurs in BTC, and the abnormal expression of glycans plays a significant role in the progression of BTC, resulting in poor survival outcomes for patients.^21^, ^22^, ^23^, ^24^, ^25^ Therefore, it was discovered that BTC‐associated glycans might be potential predictive biomarkers for diagnosis and prognosis.^24^, ^26^, ^27^ In fact, both CA50 and CA19‐9 are sialyl‐associated glycan antigens. As our study has shown, CA50 and CA19‐9 were highly correlated with each other in BTC, CHC, BBD, and HP patients. However, CA50 is rarely used clinically and few guidelines recommend CA50 in patients with BTC. Moreover, few studies have explored the diagnostic value of CA50 in combination with other tumor markers.

As previous studies reported by Haglund et al.,^20^ Luang et al.,^19^ and Watanabe et al.^28^ have shown, CA50 levels could be secreted from CCA tissues and were abnormally elevated in CCA tissues.^19^, ^20^, ^28^ However, the sample sizes of the above studies were small, and the patient data analyzed did not include GBC, VPC, CHC, and so on. To date, the exact clinical value of CA50 in BTCs remains inaccurate. Our multicenter large‐sample size study has detailed the relationship between serum CA50 levels and BTC patients, including the optimal threshold, sensitivity, specificity, accuracy, PPV, NPV, positive LR, negative LR, and AUCs with 95% CI. In this study, the levels of serum CA50, CA19‐9, and CEA in BTC patients were higher than those in non‐BTC patients, and the difference was significant. In addition, the ROC results indicated that CA50 was a potential tumor marker for the screening and diagnosis of BTC patients. Compared to AFP and CEA, serum CA50 and CA19‐9 had a better specificity and positive LR in BTCs. Moreover, the clinical diagnosis model combining CA50, CA19‐9, and AFP also showed better diagnostic performance than the single tumor marker. In addition, for the convenience of clinical use, dynamic nomogram was constructed. The clinical diagnosis model showed excellent specificity and moderate sensitivity. As a result, we considered that the diagnostic model might be more suitable for diagnosis and whether it could solve an important clinical problem, that is, the preoperative diagnosis of iCCA and HCC.

As we know, BTCs are usually classified clinically according to anatomical location. Patients with different classifications have significantly different survival prognoses and treatment strategies. In fact, it is difficult to distinguish iCCA from HCC and CHC only by imaging before obtaining pathological results.^6^ However, the choice of treatment plan depends greatly on a correct preoperative diagnosis. As a small sample size study concluded, CA50 could be a diagnostic marker candidate for iCCA.^19^ However, the control group of the above study only had BBD, HP and a few other gastrointestinal cancers and lacked many HCC and CHC patients, who were difficult to distinguish before treatment. In our study, the specificity and positive LR of serum CA50 were also higher than those of CA19‐9 and CEA in iCCA, but the sensitivity of serum CA50 was lower than that of the other markers. Considering the clinical features of CA50, we built a clinical diagnostic model again by combining CA50, CA19‐9, and AFP to help differentiate between HCC and iCCA patients. The model did not include CEA because it has a poor AUC value according to our large sample size results. The AUCs of the MODEL 2 were 0.885 in the training cohort and 0.879 in the validation cohort, which were higher than those of the single serum CA50, CA19‐9, AFP, and CEA alone. The results of DCAs also indicated that the MODEL 2 could add more benefit than the “treat none” and “treat all.” Therefore, the diagnostic model has high diagnostic value and can assist in distinguishing iCCA and HCC without relying on imaging.

In the clinic, the degree and type of pathological differentiation, tumor location, and degree of jaundice in BTC patients are all important. However, few studies have explored the correlation between different tumor markers and the clinical information of BTCs. In our study, we found that the expression levels of CA50 and CA19‐9 were higher in pCCA than in VPC, iCCA, dCCA, and GBC. We hypothesized that this difference in expression might be related to the higher incidence of obstruction of the biliary tract in pCCA. As a previous study reported by Luang S et al., in iCCA patients, patients with jaundice expressed higher levels of CA50 than those without jaundice.^19^ Therefore, we compared the levels of tumor markers in patients with different degrees of jaundice who were divided into five groups: no jaundice, recessive jaundice, mild jaundice, moderate jaundice, and severe jaundice groups. The serum levels of both CA50 and CA19‐9 increased with the severity of jaundice, and the difference was statistically significant. And CA50 was found to increase with AJCC staging. It is also known that there is a direct relationship between the degree of jaundice and tumor stage. In addition, the serum CA19‐9 levels were obviously higher in poorly differentiated adenocarcinoma than in highly differentiated adenocarcinoma in BTCs. This phenomenon may suggest that the higher the levels of CA19‐9 are, the worse the prognosis of patients. Therefore, both CA50 and CA19‐9 are not only sialyl‐associated glycan antigens but also have similar clinical features. CA50 seems to be a neglected tumor biomarker that has great clinical diagnostic value in BTCs.

However, there were still some limitations in our study. First, the study included only Asian populations, and the results may have some differences in other populations. Second, our BBD patients included few patients with primary biliary sclerosis due to retrospective limitations. Third, we did not investigate the relationship between serum markers and tumor recurrence or patient survival, which will be further explored through prospective studies. Finally, for the differential diagnosis of iCCA and HCC, imaging and pathology are necessary, and this diagnostic model can only be used as an aid to diagnosis.

## 5 CONCLUSION

To our knowledge, this is the first large‐scale multicenter study to add neglected CA50 to the clinical diagnostic model of BTCs, which has good diagnostic value in distinguishing iCCA from HCC by combining CA50, CA19‐9, and AFP.

## AUTHOR CONTRIBUTIONS

**Yong‐Shuai Wang:** Conceptualization (lead); data curation (supporting); writing – original draft (equal). **Wei Wang:** Formal analysis (equal). **Shen‐Yu Zhang:** Formal analysis (equal). **Wei Cai:** Formal analysis (equal); validation (equal). **Rui‐Peng Song:** Visualization (equal). **Tao Mei:** Data curation (equal). **Wei Wang:** Methodology (equal). **Feng Zhang:** Project administration (equal). **Fei‐Yu Qi:** Data curation (equal); formal analysis (equal). **Sai Zhang:** Data curation (equal). **Yan Liu:** Formal analysis (equal); investigation (equal). **Hao‐Ran Li:** Formal analysis (equal). **Peng Ji:** Visualization (equal). **Miao Gao:** Formal analysis (equal). **Hua‐Chuan Song:** Formal analysis (equal). **Huan‐Zhang Yao:** Investigation (equal). **Fan‐Zheng Meng:** Data curation (equal). **Zheng Lu:** Funding acquisition (equal). **Ji‐Zhou Wang:** Supervision (equal); writing – original draft (equal); writing – review and editing (equal). **Lian‐Xin Liu:** Funding acquisition (equal); writing – review and editing (equal).

## FUNDING INFORMATION

This work was partly supported by the National Natural Science Foundation of China (grant number 82170618).

## CONFLICT OF INTEREST STATEMENT

No conflict of interest exists in the submission of this manuscript.

## Supporting information


Figure S1.

Figure S2.

Figure S3.

**Tables S1–S4**.

## Data Availability

The data generated in this study are available upon request from the corresponding author.
