# Supplementary figures and images for: Diagnostic value of carbohydrate antigen 50 in biliary tract cancer: A large‐scale multicenter study
Source: Cancer Med. 2024 Jun 26;13(12):e7388. doi: 10.1002/cam4.7388 (PMC11200271; doi:10.1002/cam4.7388)

Supplementary Figure 1

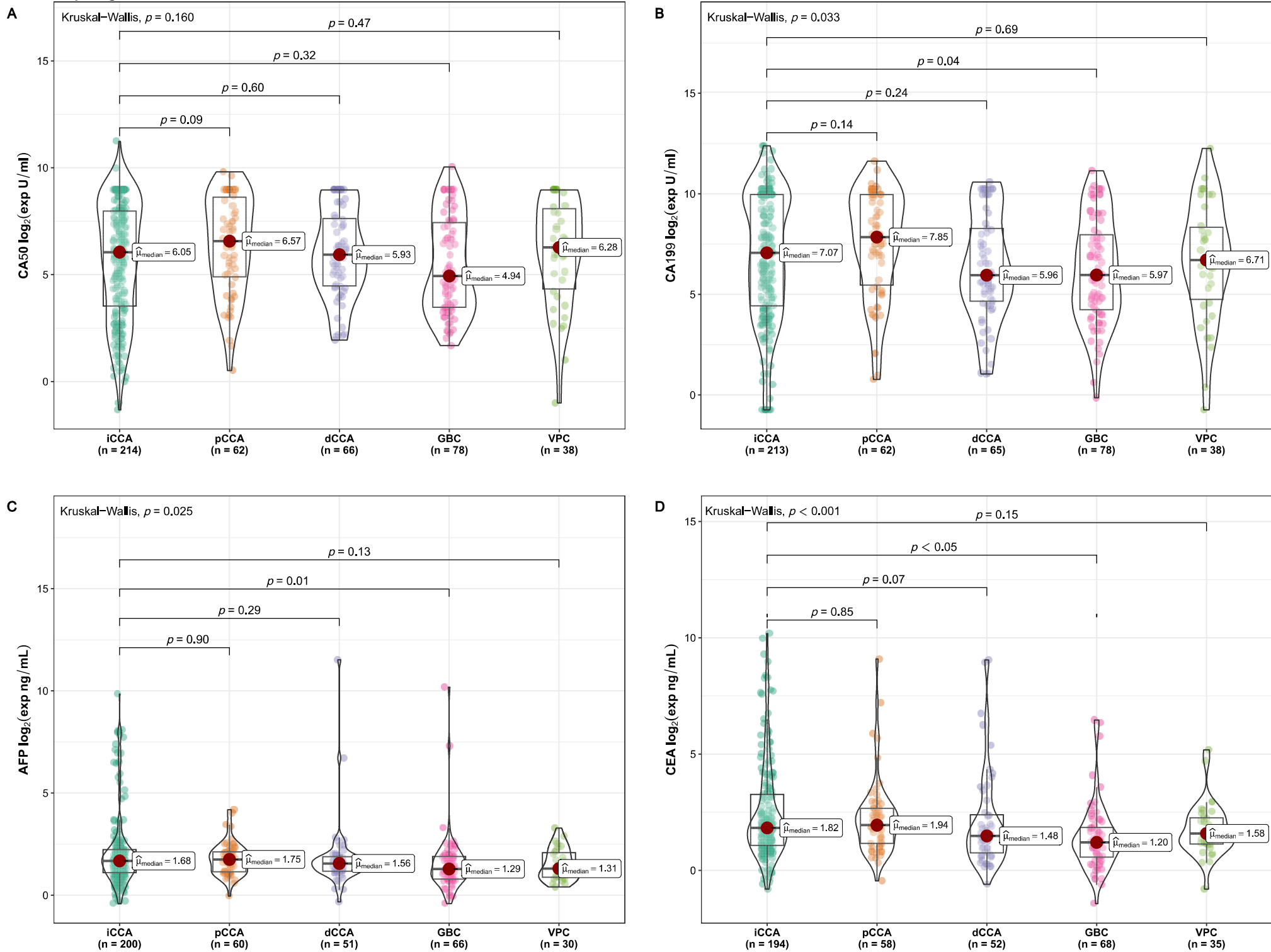

Supplement: Supplementary file 1 — Figure S1. [file CAM4-13-e7388-s003.pdf]

Supplementary Figure 2

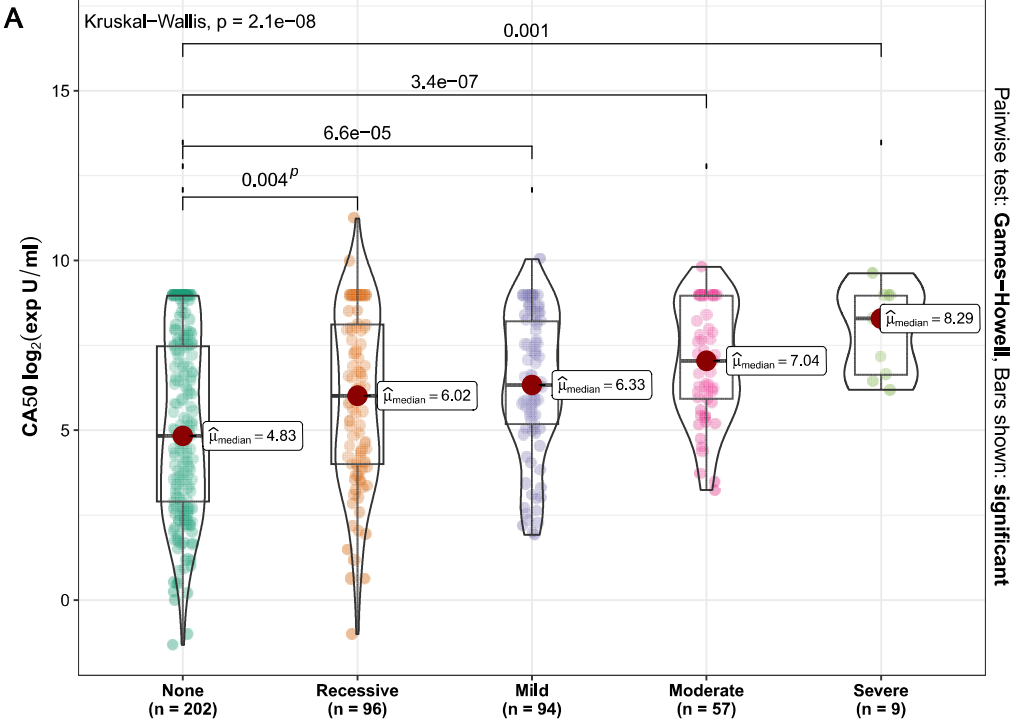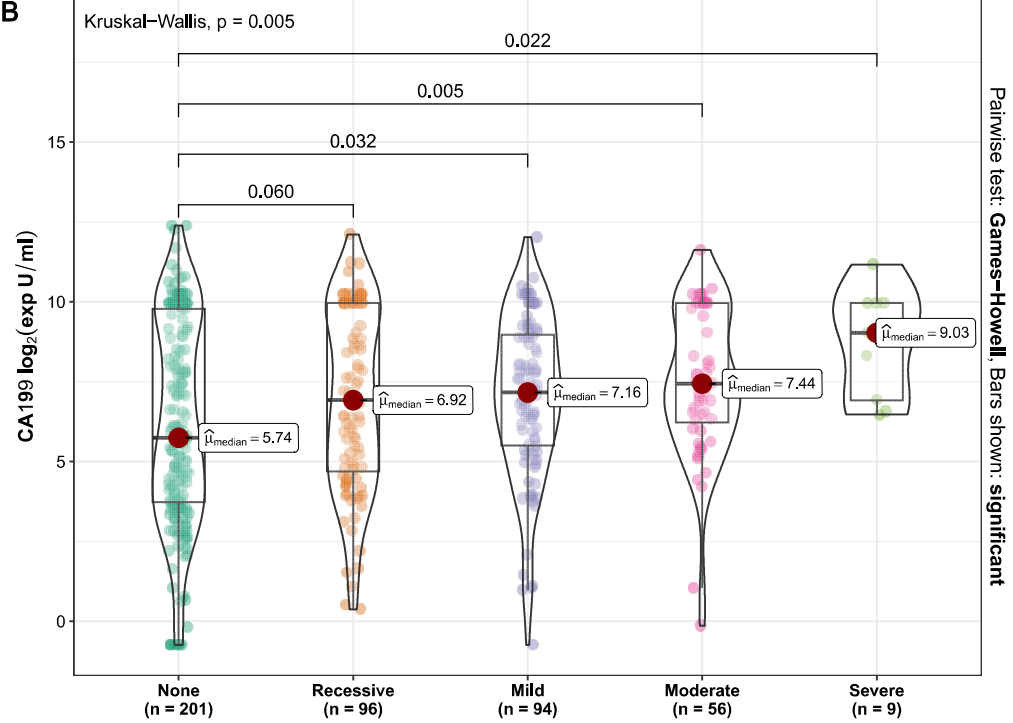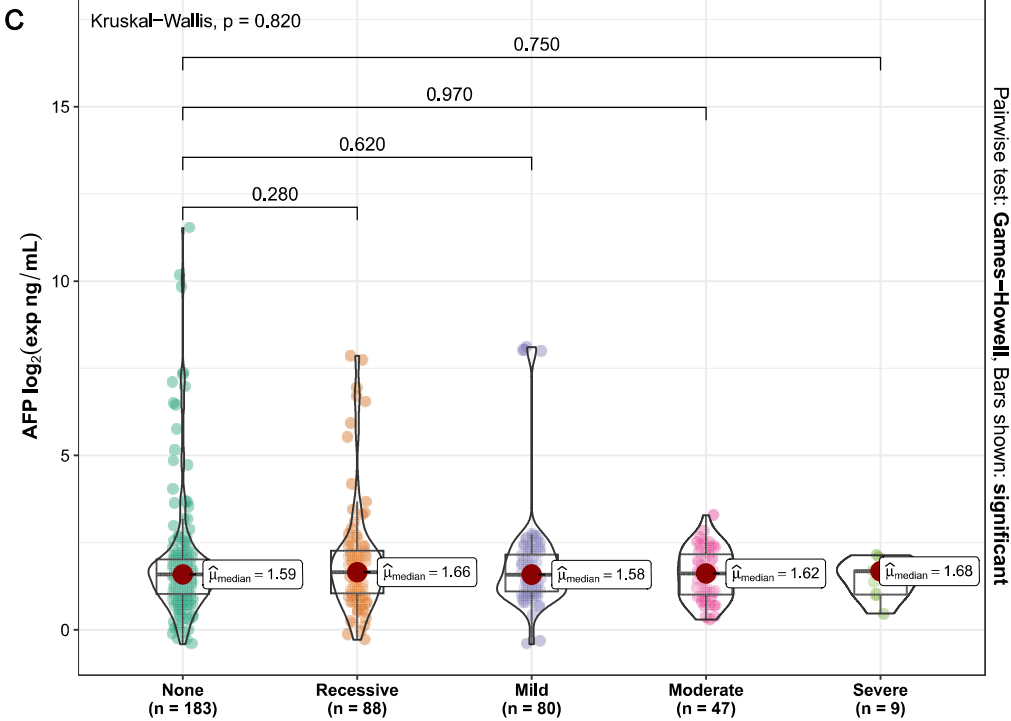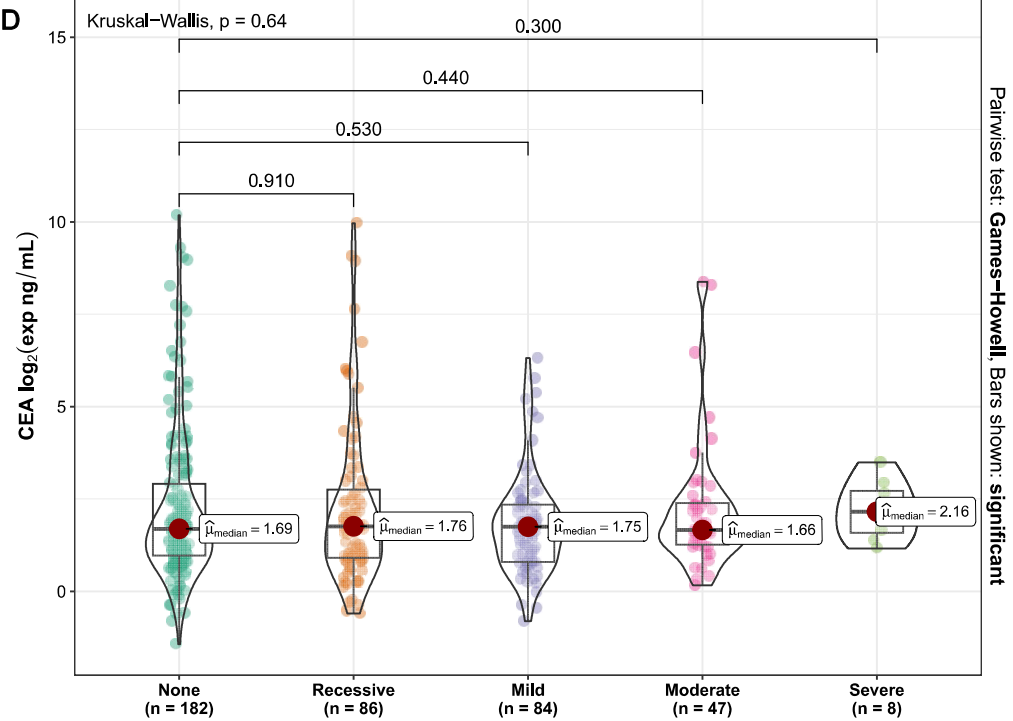

Supplement: Supplementary file 2 — Figure S2. [file CAM4-13-e7388-s001.pdf]

Supplementary Figure 3

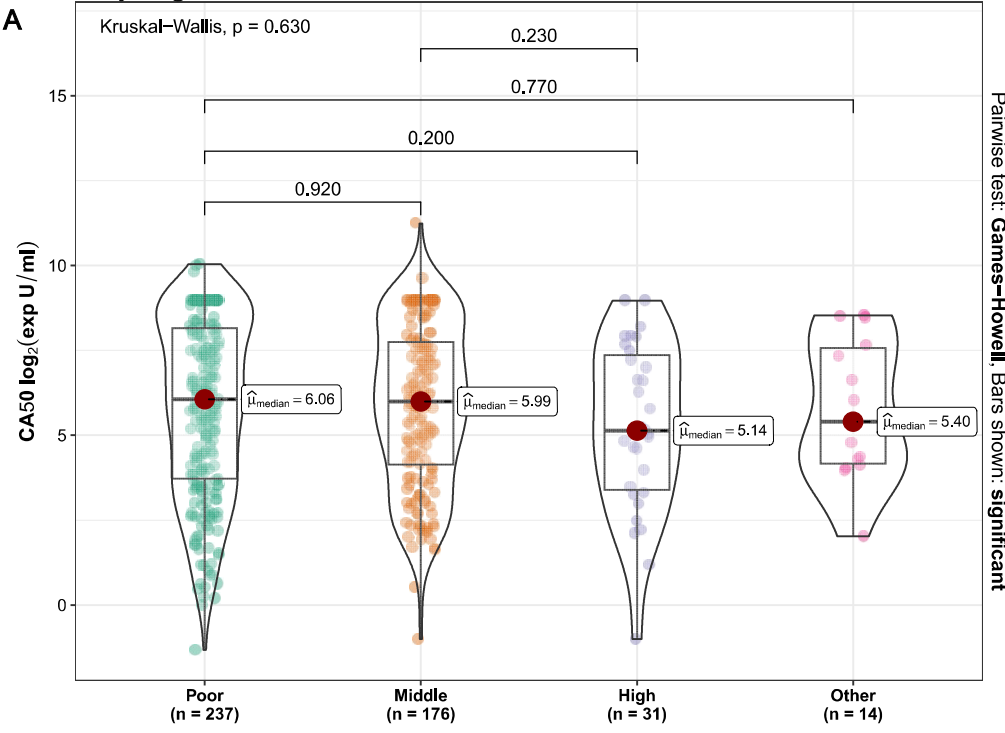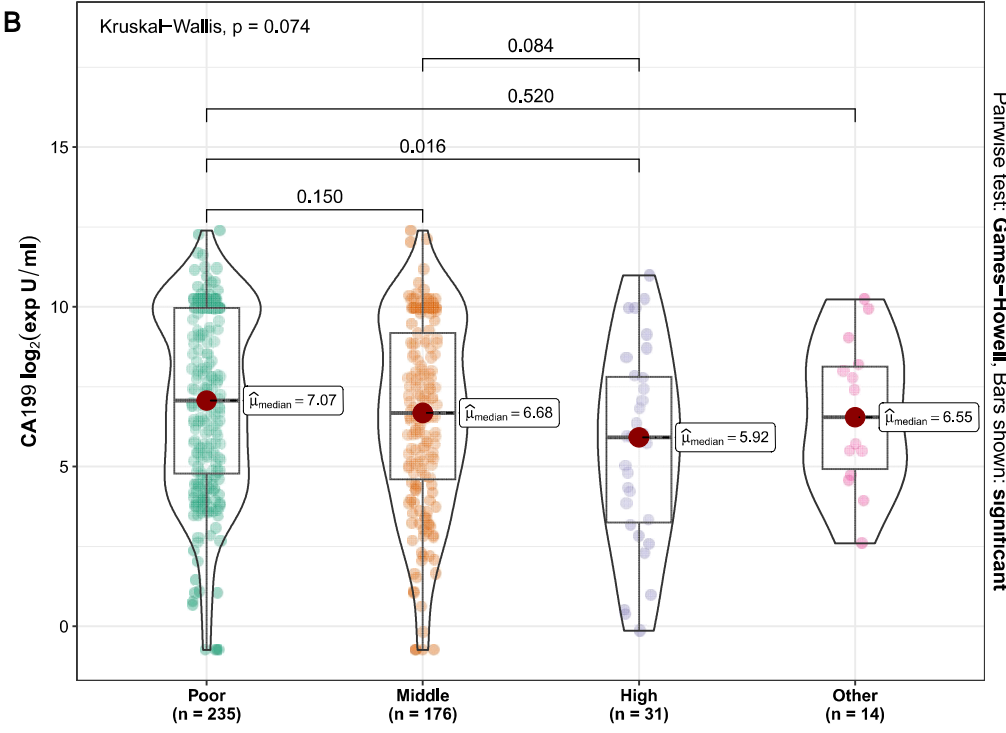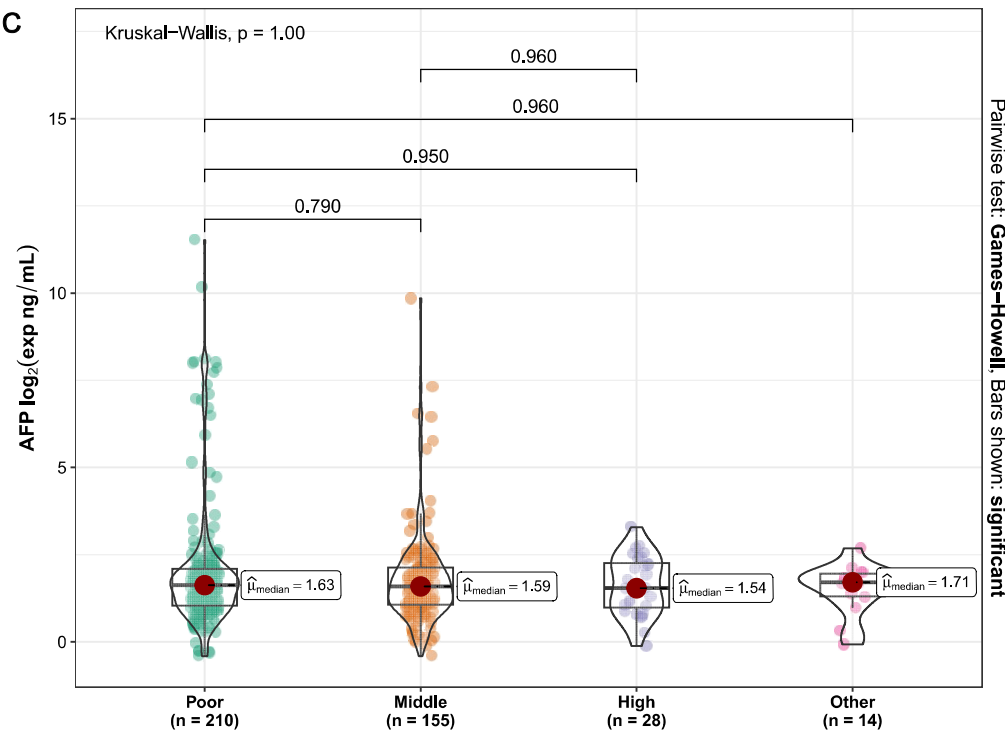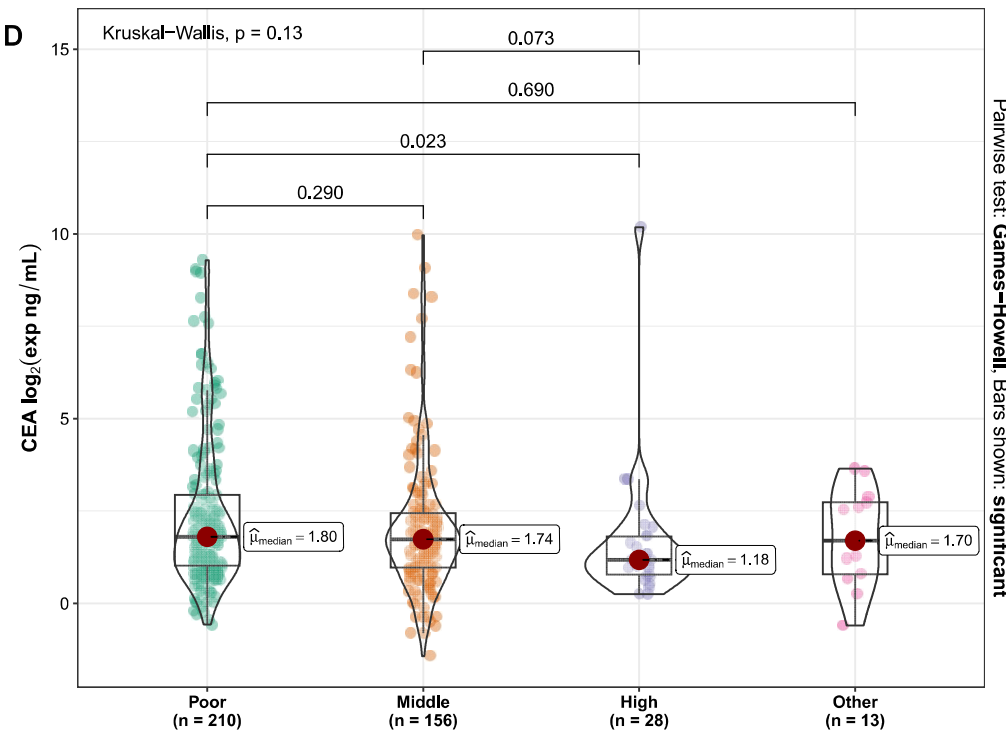

Supplement: Supplementary file 3 — Figure S3. [file CAM4-13-e7388-s002.pdf]
