# Supplementary material for: Diagnostic value of carbohydrate antigen 50 in biliary tract cancer: A large‐scale multicenter study
Source: Cancer Med. 2024 Jun 26;13(12):e7388. doi: 10.1002/cam4.7388 (PMC11200271; doi:10.1002/cam4.7388)
Supplement: Supplementary file 4 — Tables S1–S4. [file CAM4-13-e7388-s004.docx]

**Supplementary Table 1. Levels of tumor markers in different locations of BTC.**

|  | iCCA  (n =214） | pCCA  (n =62) | dCCA  (n =66) | GBC  (n =78) | VPC  (n =38) | *P* |
| --- | --- | --- | --- | --- | --- | --- |
| Median CA50 (min, mix), U/ml | 66.5 (0.4-2412.2) | 95.3 (1.4-899.5) | 61.3 (3.8-500.0) | 30.9 (3.2-1050.7) | 77.7 (0.5-500.0) | 0.104 |
| Median CA19-9 (min, mix), U/ml | 134.4 (0.6-5355.0) | 231.9 (1.7-3163.9) | 62.1 (2.1-1534.0) | 62.5 (0.9-2271.0) | 104.8 (0.6-4882.0) | 0.020 |
| Median AFP (min, mix), ng/mL | 3.2 (0.8-920.6) | 3.4 (1.0-18.2) | 2.9 (0.8-2933.0) | 2.4 (0.8-1161.0) | 2.5 (1.3-9.8) | 0.026 |
| Median CEA (min, mix), ng/mL | 3.5 (0.6-1160.3) | 3.8 (0.7-543.0) | 2.8 (0.7-528.7) | 2.3 (0.4-88.3) | 3.0 (0.6-36.2) | 0.001 |

Note: biliary tract cancer (BTC); intrahepatic cholangiocarcinoma (iCCA); perihilar cholangiocarcinoma (pCCA); distal cholangiocarcinoma (dCCA); vater ampulla carcinoma (VPC); carcinoma of gallbladder (GBC); carbohydrate antigen 50 (CA50); carbohydrate antigen 19-9 (CA19-9); α-fetoprotein (AFP); carcinoembryonic antigen (CEA).

**Supplementary Table 2. Levels of tumor markers in BTC patients with different degrees of jaundice.**

|  | No jaundice  (n =202) | Recessive jaundice  (n =96) | Mild jaundice  (n =94) | Moderate jaundice  (n =57) | Severe jaundice  (n =9) | *P* |
| --- | --- | --- | --- | --- | --- | --- |
| Median CA50 (min, mix), U/ml | 28.5 (0.4-500.0) | 65.3 (0.5-2412.2) | 80.7 (3.8-1050.7) | 132.0 (9.5-899.5) | 312.8 (73.3-789.8) | <0.001 |
| Median CA19-9 (min, mix), U/ml | 53.6 (0.6-5355.0) | 121.4 (1.3-4421.0) | 143.6 (0.6-4179.0) | 173.8 (0.9-3163.9) | 522.9 (88.9-2298.0) | 0.010 |
| Median AFP (min, mix), ng/mL | 3.0 (0.8-2933.0) | 3.2 (0.8-231.8) | 3.0 (0.8-274.6) | 3.1 (1.2-9.8) | 3.2 (1.4-4.4) | 0.721 |
| Median CEA (min, mix), ng/mL | 3.2 (0.4-1160.3) | 3.4 (0.7-1000.0) | 3.4 (0.6-79.6) | 3.2 (1.1-331.7) | 4.5 (2.2-11.3) | 0.583 |

Note: carbohydrate antigen 50 (CA50); carbohydrate antigen 19-9 (CA19-9); α-fetoprotein (AFP); carcinoembryonic antigen (CEA).

**Supplementary Table 3. Levels of tumor markers in BTC patients with different degrees of pathological differentiation.**

|  | Poorly differentiated adenocarcinoma  (n =237) | Moderately differentiated adenocarcinoma  (n =176) | Highly differentiated adenocarcinoma  (n =31) | Other types ^a^  (n =14) | *P* |
| --- | --- | --- | --- | --- | --- |
| Median CA50 (min, mix), U/ml | 66.6 (0.4-1050.7) | 63.8 (0.5-2412.2) | 35.2 (0.5-500.0) | 46.3 (4.1-369.0) | 0.358 |
| Median CA19-9 (min, mix), U/ml | 134.4 (0.6-5355.0) | 102.8 (0.6-5353.0) | 60.4 (0.9-2021.0) | 110.1 (6.1-1200.0) | 0.048 |
| Median AFP (min, mix), ng/mL | 3.1 (0.8-2933.0) | 3.0 (0.8-920.6) | 2.9 (0.9-9.8) | 3.3 (0.9-6.4) | 0.795 |
| Median CEA (min, mix), ng/mL | 3.5 (0.7-624.8) | 3.3 (0.4-1000.0) | 2.3 (1.2-1160.3) | 3.2 (0.7-12.5) | 0.294 |

Note: carbohydrate antigen 50 (CA50); carbohydrate antigen 19-9 (CA19-9); α-fetoprotein (AFP); carcinoembryonic antigen (CEA);

Other types ^a^ include squamous cell carcinoma, adeno-squamous carcinoma, neuroendocrine carcinoma and so on.

**Supplementary Table 4. Levels of tumor markers in BTC patients with different AJCC staging.**

|  | AJCC 0-1 staging  (n =316) | AJCC 2 staging  (n =100) | AJCC 3-4 staging  (n =42) | *P* |
| --- | --- | --- | --- | --- |
| Median CA50 (min, mix), U/ml | 58.6 (0.4-2412.2) | 74.9 (1.8-500.0) | 182.8 (2.33-500.0) | 0.037 |
| Median CA19-9 (min, mix), U/ml | 96.9 (0.6-5355.0) | 169.8 (0.6-4882.0) | 223.4 (4.6-12000.0) | 0.171 |
| Median AFP (min, mix), ng/mL | 3.1 (0.8-2933.0) | 3.1 (0.8-260.1) | 2.8 (0.8-260.1) | 0.619 |
| Median CEA (min, mix), ng/mL | 3.3 (0.6-1160.3) | 3.2 (0.4-624.8) | 3.2 (0.7-505.5) | 0.070 |

Note: carbohydrate antigen 50 (CA50); carbohydrate antigen 19-9 (CA19-9); α-fetoprotein (AFP); carcinoembryonic antigen (CEA); Other types ^a^ include squamous cell carcinoma, adeno-squamous carcinoma, neuroendocrine carcinoma and so on.
